# Novel and characteristic radiological features of neurosyphilis: a case series

**DOI:** 10.1186/s12883-024-03762-5

**Published:** 2024-07-20

**Authors:** Kenji Ohira, Nanako Hashimoto, Daisuke Kanai, Yukio Inoue

**Affiliations:** 1 Department of Radiology, Japanese Red Cross Society Shizuoka Hospital, 8-2 Otemachi, Aoi Ku, Shizuoka City, Shizuoka Prefecture 420- 0853 Japan; 2 Department of Radiology, Chutoen General Medical Center, 1-1 Shobudaike, Kakegawa City, Shizuoka Prefecture 436-8555 Japan

**Keywords:** Syphilis, Neurosyphilis, Radiology, Otosyhilis, Osteosyphilis, Syphilitic optic neuritis, *Treponema pallidum*, Case series

## Abstract

**Background:**

*Treponema pallidum* can invade the central nervous system (CNS) early in its infection, causing neurosyphilis. Neurosyphilis typically presents with meningovasculitis in the acute or subacute phase, while tabes dorsalis and dementia paralytica are classical conditions in the later stages. However, syphilis is often misdiagnosed as other conditions such as tumors or autoimmune diseases including vasculitis and encephalitis, which is why the condition is known as “The Great Mimicker.” The increasing incidence of syphilis in recent years emphasizes the importance of early diagnosis and treatment; however, its multiple clinical manifestations impose diagnostic challenges for clinicians because it resembles other diseases. In this case series, we present the impressive manifestations of neurosyphilis through three unique radiological presentations.

**Case presentation:**

Case 1 details optic nerve involvement in an HIV-positive male, where MRI and fundoscopic findings confirmed syphilitic optic neuritis. Case 2 describes a patient in her pregnancy initially suspected of acoustic neuroma on MRI, later diagnosed with syphilitic gumma affecting the inner ear canal. Case 3 is a young male with clinical features mimicking temporal arteritis, ultimately identified as skull osteomyelitis secondarily causing inflammation of the musculus temporalis and meningitis.

**Conclusions:**

These cases underscore the necessity of considering syphilis in differential diagnoses, given the diversity of its clinical presentations. Radiology plays an important role in avoiding unnecessary interventions. The increasing prevalence of recurrent syphilis imposes diagnostic challenges, emphasizing the importance of the early diagnosis and treatment of neurosyphilis by clinicians.

## Background

Syphilis is an infectious disease caused by *Treponema pallidum*. This condition is categorized into primary, secondary, latent, and tertiary syphilis. It was previously posited that neurosyphilis predominantly manifested during the tertiary phase of the disease. However, it has been speculated that *Treponema pallidum* invades the central nervous system through hematogenous route from the infection site, thereby making it possible for the pathogen to progress in the central nervous system (CNS) even in the early phase of the disease [1]. As per the guidelines from the Centers for Disease Control and Prevention (CDC), the diagnostic standard for syphilis includes blood serological tests alongside clinical features [2]. Confirmation of syphilis requires at least one nontreponemal antigen test along with one treponemal antigen test. The Venereal Disease Research Laboratory and Rapid Plasma Reagin (RPR) tests are the most widely used and recommended nontreponemal antigen tests [3]. Treponemal antigen tests, also known as specific tests such as the *Treponema pallidum* haemagglutination assay (TPHA), the Fluorescent Treponemal Antibody Absorption test (FTA-ABS), and the *Treponema pallidum* Particle Agglutination test, are widely utilized [2]. The diagnosis of neurosyphilis generally requires a combination of epidemiological data, neurologic or neuropsychiatric symptoms, serological analyses of blood and cerebrospinal fluid (CSF), and imaging studies such as MRI in certain cases. Generally, the major guidelines recommend the patients with syphilis presented with neurological symptoms to undergo a lumbar puncture [4, 5].

In this paper, we present three atypical presentations of neurosyphilis; however, these cases showed characteristic radiographic findings. MRI findings are subtle or difficult to identify, even for experienced neurologists and radiologists. Therefore, neurosyphilis is often called “The Great Imitator” [6]. Single-photon emission computed tomography using echnetium-99m ethyl cysteinate dimer (99mTc-ECD) was used in a recent study to test functional abnormalities in neurosyphilis because they are hard to detect on MRI [7].

The first case was that of an HIV-positive male in his thirties who had optic nerve syphilis. The clinical manifestations of the condition included papillary edema that was detected by an orbital examination and optic disc protrusion, which was observed on brain MRI. However, MRI findings regarding neurosyphilis with ocular involvement are often considered normal for ophthalmologists [8], which implies that neurosyphilis with ocular involvement might be overlooked because of the lack of precise MRI interpretation of optic nerve lesions without experienced neurologists or radiologists. It has been reported that 65% of patients with ocular syphilis are coinfected with human immunodeficiency virus (HIV). Therefore, it is important to consider the possibility of syphilitic optic neuropathy when HIV-positive patients present with optic neuropathy symptoms [9]. The second case was that of a pregnant woman in her twenties who presented with right facial paralysis and dizziness. MRI showed an internal auditory canal mass, which was initially suspected to be neurinoma but turned out to be syphilitic gumma. The third case was that of a young male in his twenties who presented with fever and headache. He was clinically suspected to have temporal arteritis based on the clinical manifestations and serological tests. However, MRI revealed several hyper-signal-intensity lesions on T2-weighted images (T2WI) and enhancement on postcontrast T1-weighted images (T1WI) in the calvaria. His final diagnosis was osteomyelitis of the skull involving the temporalis muscle. Also, contrast enhancement on the dura mater and dural thickening was also shown, suggesting meningitis secondary to osteomyelitis. Although the unavailability of standardized diagnostic criteria in radiology regarding atypical presentations of neurosyphilis makes the early diagnosis more difficult, to the best of our knowledge, this is the first atypical neurosyphilis case series involving confirmation through MRI. This case series could be helpful for future neurosyphilis diagnosis since syphilis is a global pandemic. Moreover, the acute rise in the incidences of coinfections of syphilis and HIV has become a global problem. Research has demonstrated that patients with syphilis who are coinfected with HIV are more predisposed to developing neurosyphilis. Additionally, syphilis coinfected with HIV can show atypical clinical manifestations, and can progress to neurosyphilis even in the early stages of infection [10].

## Case presentations

### Case 1

**Fig. 1:**
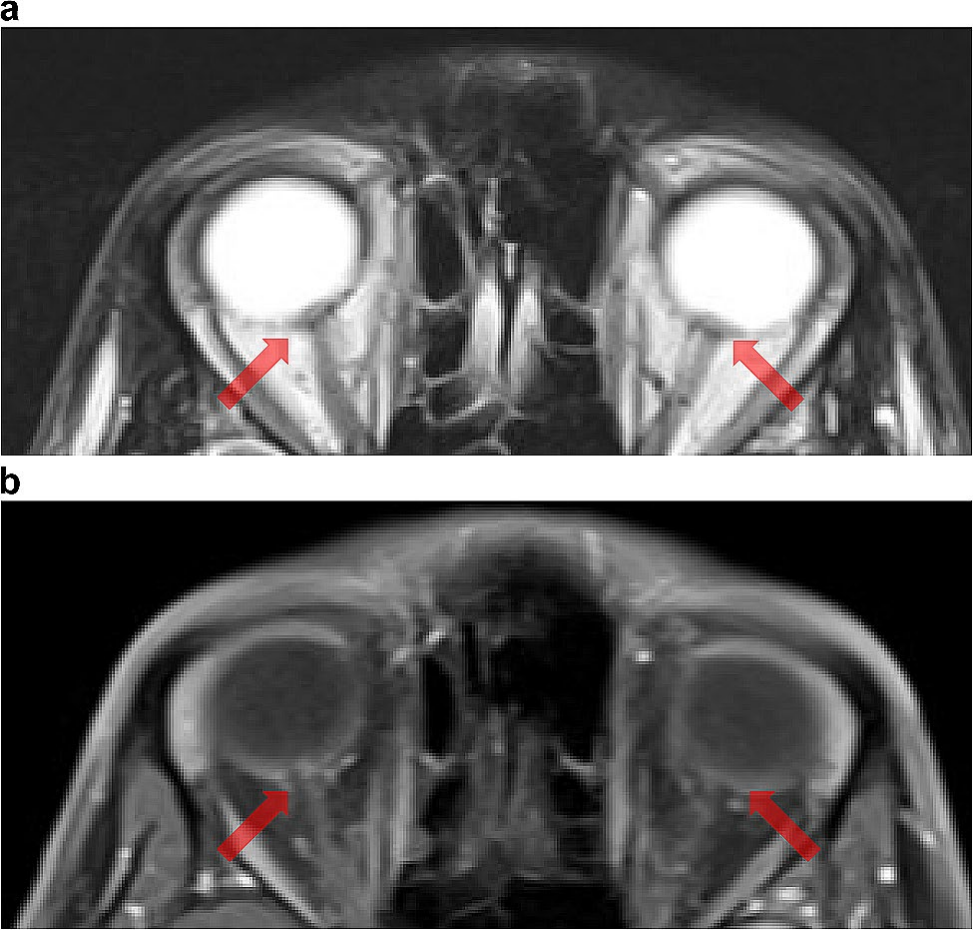
T2WI (**a**) showing protrusions of both optic discs (arrows). (**b**) There was no enhancement in the optic nerve or disc on postcontrast T1WI

A male patient in his thirties who has been infected with HIV presented with visual disturbances such as blurred and foggy vision. Three years ago, the patient was diagnosed with HIV and commenced highly active antiretroviral therapy (HAART). However, the patient demonstrated poor adherence to treatment. Approximately a year ago, the patient independently discontinued the HAART and outpatient visits. Ophthalmological examinations found optic disc edema. His bilateral visual acuity score of 20/20 was decreased to 6/20 on the eye test. The physical examination performed revealed no significant abnormalities. Additionally, no significant neurological abnormalities were detected, except visual acuity loss. Blood tests revealed that the CD4^+^ lymphocyte count was 215/µL and the viral load was 27,400 copies/mL. Serological RPR and the TPHA revealed positive titers (78.2R.U. and 14,183T.U., respectively). CSF analyses revealed an initial pressure of 15 cm H_2_O, a cell count of 128 (comprising 98% mononuclear cells), and a protein level of 99 mg/dL, which is suggestive of meningitis. Also, CSF analyses indicated an RPR of 3.2R.U and TPHA at a dilution of 1 in 10,240. A brain MRI (Fig. 1) revealed bilateral protrusions of the optic disc. Postcontrast T1WI did not demonstrate any significant enhancement in the optic nerve or disc. Additionally, no increased signal on T2WI nor contrast enhancements were observed within the brain parenchyma. MRI did not reveal any classical signs of meningitis, such as thickening or contrast enhancement of the dura. His final diagnosis was syphilitic optic neuritis. Since Penicillin G administration was started, his symptoms have improved quickly.

### Case 2

**Fig. 2:**
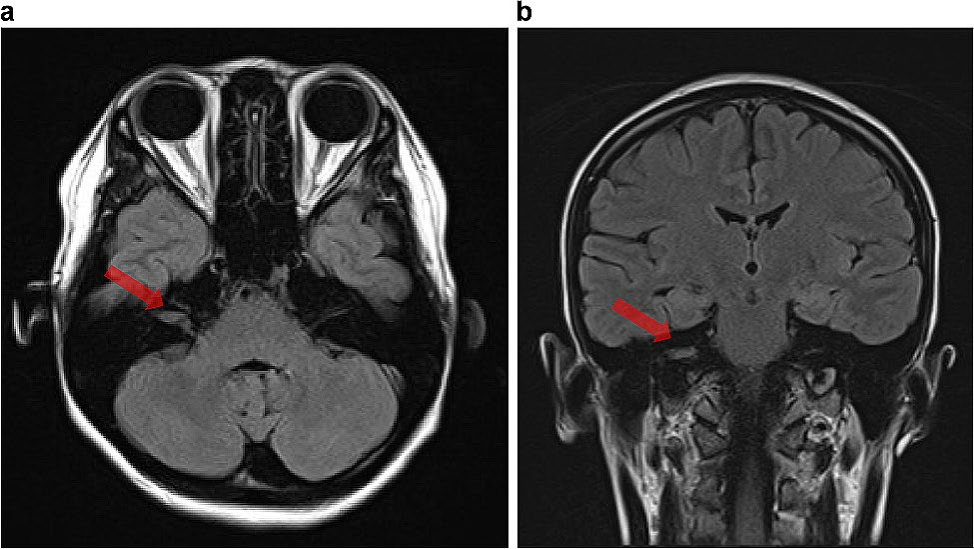
A mass (arrow) in the right auditory canal on axial (**a**) and coronal (**b**) FLAIR.

A female pregnant patient in her twenties visited an otolaryngologist complaining of enlarged right posterior cervical lymph nodes. A sensorineural hearing loss in the right ear was detected during the medical examination. Right facial paralysis and vertigo occurred one month after her initial visitation, so she came to the emergency room of our hospital. The physical examination performed revealed right cervical lymphadenopathy and no other notable abnormalities. Right-sided deafness and facial paralysis were observed during the neurological assessment that was performed. Other cranial nerve functions were considered normal. Blood tests were positive for PRP (847R.U.) and TPLA (307T.U.), and no other abnormalities (including evidence of HIV infection) were identified. CSF analyses were not conducted as an otolaryngologist diagnosed her with peripheral vertigo. A brain MRI was performed to exclude an acute cerebral infarction because of her acute-onset dizziness. The images (Fig. 2) revealed a mass in the right auditory canal, which the otolaryngologist considered an acoustic neuroma. However, otosyphilis due to gumma was radiologically considered given the results of the serological tests, including positive PRP and TPLA results. The patient was presumed to have stage II syphilis considering the local lymph node enlargement. Subsequently, treatment with Penicillin was initiated, resulting in the improvement of her symptoms. After treatment, the patient successfully gave birth to a child with no evidence of congenital syphilis. Furthermore, the infant demonstrated normal growth and no neurological developmental delay. According to the disease course, her final diagnosis was otosyphilis caused by acoustic gumma.

### Case 3

**Fig. 3:**
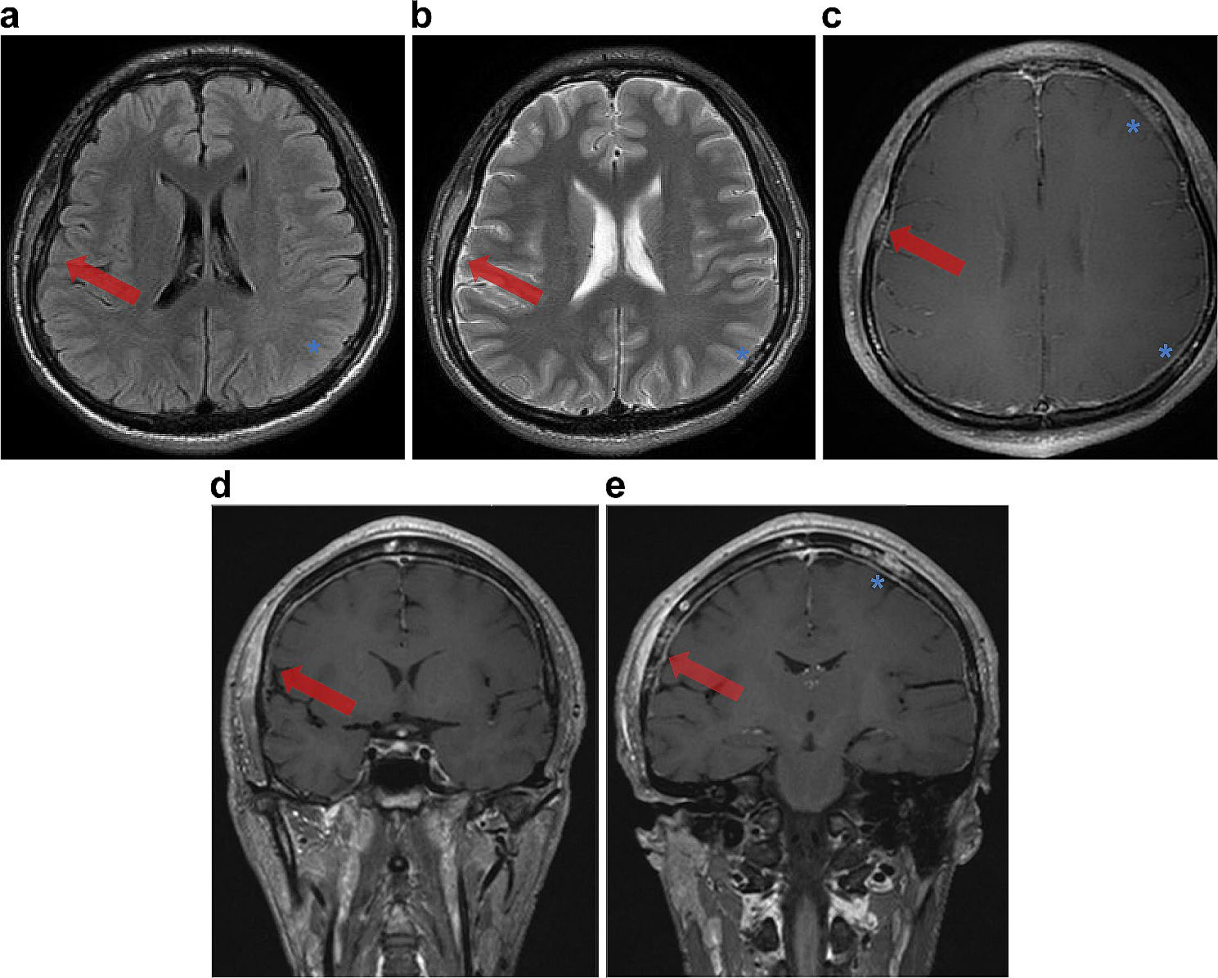
Hyperintense lesion (arrow) on T2WI (**a**) and FLAIR (**b**) in the right temporal bone with contrast enhancement on postcontrast T1WI (**c**, **d**, **e**), which is indicative of osteomyelitis. Adjacent soft tissues (including the temporalis muscle) were involved with swelling and hyperintensity on T2WI (**a**) and FLAIR (**b**) and enhancement on postcontrast T1WI (**c**, **d**, **e**). There were several hyperintense lesions on T2WI and FLAIR, and also contrast-enhanced lesions in the skull base. Dural enhancement was also observed (**c**, **d**, **e**), which is suggestive of meningitis

A male in his twenties was referred to the hospital with complaints of headache and fever. Physical examination revealed tenderness in the right temporal region. Laboratory tests revealed a white blood cell count of 7,100/µL and a C-reactive protein level of 11 mg/dL, which are suggestive of an inflammatory disease. Temporal arteritis was suspected given his clinical symptoms and inflammatory process as well as an elevated erythrocyte sedimentation rate of 119 mm/h. A brain MRI with gadolinium contrast enhancement was performed for further evaluation. MRI (Fig. 3) showed multiple hyperintense lesions on T2WI and fluid-attenuated inversion recovery (FLAIR) in the cranial bones and contrast enhancement in the cranial bones was observed on postcontrast T1WI, which findings were suggestive of osteomyelitis. Lesions extending from the right temporal bone into the surrounding soft tissues involved the right temporalis muscle causing myositis. Combining these findings, myositis was considered a complication of osteomyelitis, rather than temporal arteritis which was the primary diagnosis. A brain MRI also indicated dural enhancement suggestive of meningitis mainly observed near the bone lesions. Serologic tests yielded positive results for RPR (102R.U.) and TPLA (302T.U.), confirming that his final diagnosis was osteosyphilis. CSF analyses revealed that the patient’s glucose level was 50 mg/dL, protein level was 77 mg/dL, leukocyte count was 139/mm³, RPR was 4.7R.U, and TPHA dilution at 1 in 2,560. He tested negative for HIV. The detection of roseola syphilitica across body regions, alongside specific cranial bone lesions, confirmed the diagnosis of secondary syphilis with myositis of the temporalis muscle and meningitis secondary to osteosyphilis. Penicillin treatment was administered, resulting in the improvement of his symptoms without any aftereffects.

## Discussion and conclusions

The incidence of syphilis has been on the rise in recent years. Syphilis is known as the “great masquerader.” because its clinical presentation mimics those of other diseases. We presented three atypical cases of neurosyphilis, imposing significant challenges in the diagnosis of this disease. The summarized clinical data are shown in Table 1.

**Table 1:** Patient demographics and clinical findings

	age	sex	symptom	HIV infection	MRI findings	diagnosis
Case 1	30s	male	visual loss	positive	optic disk protrusions	optic neuritis
Case 2	20s	female	hearing loss facial paralysis	negative	gumma in the right auditory canal	otosyphilis
Case 3	20s	male	fever headache	negative	osteosyphilis myositis of the temporal muscle dural enhancement	osteosyphilis

The patient in Case 1 was initially diagnosed with syphilitic optic neuritis. Ocular impairment occurs in more than 3% of cases of syphilis [11, 12], and it might be an initial infectious site of neurosyphilis [13]. The involvement of the optic nerve, which presents as papilledema, optic perineuritis, or optic neuritis, is the second common manifestation of syphilitic ocular impairment [14]. Fundoscopic examination of syphilitic optic neuritis typically reveals swelling of the optic disc, affecting at least one eye [15].

Although there are only a few reports on radiologic findings in optic nerve syphilis [15–17], they are mainly categorized into two types: one in which lesions are confined to the optic disc and/or the transitional zone between the optic disc and optic nerve and the other in which inflammation is observed in the optic nerve sheath (dura mater), which is indicative of perineuritis.

In the first type, optic disc protrusion and mild optic nerve swelling with slight or no enhancement after gadolinium contrast enhancement are observed on MRI [15, 16]. There was a projection of the optic disc without contrast enhancement on MRI in the present case, which is consistent with the findings described in the literature [15, 16]. The latter type exhibits inflammation of the optic nerve sheath (the dura mater). MRI reveals significant contrast enhancement in the optic nerve sheath [17]. Syphilis progresses through three stages: primary, secondary, latent, and tertiary [18]. Ocular involvement can occur during any of the above stages and cause blindness if left untreated [18]. Optic neuritis caused by syphilis imposes significant diagnostic challenges. A previous study revealed that approximately 40% of patients who experienced visual loss due to this infection were misdiagnosed at their initial presentation. Furthermore, visual loss was not fully recovered in approximately 40% of these cases [19].

Cat-scratch disease and giant cell arteritis (GCA) are differential diagnoses to consider when evaluating syphilitic optic neuritis. MRI reveals optic disc protrusion, and contrast enhancement which is a characteristic feature of cat-scratch disease [20] that was not seen in syphilitic optic neuritis. This distinction in image findings helps in differentiating cat-scratch disease from syphilis. Furthermore, the presence of a “central bright spot sign” on contrast-enhanced MRI images is a distinctive feature of giant GCA [21]. This sign is not observed in optic nerve syphilis, which is helpful in distinguishing GCA from optic nerve syphilis.

The number of patients coinfected with syphilis and HIV have increased and they are more prone to develop neurosyphilis [22]. One possible explanation for this is that CNS has an immune privilege due to the blood–brain barrier (BBB) and limits immune cell movement. However, in HIV-positive patients, the CD4 T cell count reduction and the presence of meningeal lesions compromise the ability of CNS to defend against *Treponema pallidum* invasion [23]. Neurosyphilis coinfected with HIV is prevalent in younger patients as in this case. In addition, optic nerve symptoms are commonly associated with syphilis and HIV coinfection. Studies have reported that patients with a higher viral load and a lower CD4(+) T cell count (< 350 cells/µL) have higher risk of developing neurosyphilis compared with the general population [24, 25]. *T. pallidum* can induce the direct programmed cell death of CD4(+) cells, which contributes to an increase in the HIV-RNA viral load [26, 27]. Also, it is well reported that the absence of HAART increases the risk of developing neurosyphilis [24, 25, 28].

In second case, which was related to inner ear syphilis otosyhpilis, the patient presented with facial paralysis, hearing loss, and dizziness. In this case, the etiology was an auditory canal gumma.

Two potential mechanisms have been proposed for otosyphilis. First, the syphilitic meningitis affects the seventh and eighth cranial nerves, which causes neuritis as a course of meningitis and second, the TP directly invades the inner ear via the bloodstream, which leads to the inner ear inflammation [29, 30].

A previous report [31] documented that gadolinium contrast-enhanced MRI can show the auditory nerve, facial nerve, cochlea, and vestibule. Auditory symptoms are frequently observed in the early latent, late latent, or tertiary stage of syphilis; however, such symptoms are observed even in early syphilis, which means that otosyphilis can exist at any stage in the course of the disease.

The current case exhibits a syphilitic gumma confined to the internal auditory canal. While gumma typically appears later in the disease course except the CNS, cerebral syphilitic gumma is formed earlier than the trunk and extremities gumma during the disease course. HIV-positive patients tend to develop CNS gumma earlier than HIV-negative patients [32].

To our knowledge, there has been only one study reporting gumma localized to the internal auditory canal [33]. Our case showed no evidence of meningeal involvement like the one in the previous report17], suggesting that CNS gumma is not invariably associated with meningeal lesions. It is also important to highlight that the patient in our case was not HIV-positive, indicating that syphilitic gummas can occur in the early stages even in the absence of HIV infection.

The final case was that of osteosyphilis of the skull involving the temporalis muscle, presenting symptoms resembling those of temporal arteritis. Additionally, the MRI displayed meningitis mostly adjacent to the syphilis-induced osteomyelitis.

Syphilitic bone involvement is usually observed in newborns (transplacental infection) with syphilis-infected mothers as congenital syphilis or in the later stages of the disease as acquired syphilis. A retrospective study has revealed that bone lesions occur in only 0.15–0.23% of adult patients with early-stage syphilis [34]. Although it is quite rare, osteosyphilis in secondary syphilis typically presents 4–10 weeks following the initial syphilis infection [35, 36]. Exhibiting roseola syphilitica, the patient in the presented case was also considered to have a secondary syphilitic infection.

According to this rarity and syphilis being known as the “great imitator,” even highly experienced clinicians could have misdiagnosed these lesions as other diseases. Thus, radiologic findings play an important role in accurately diagnosing osteosyphilis, especially considering that the image quality of CT and MRI has improved and the actual incidence of the disease would be supposed to rise given the increasing prevalence of the infection.

A previous study [37] revealed that the long bones of the limbs in 22 cases were the most frequently involved in this study, closely followed by skull involvement in 21 cases and other affected bones including the ribs (5 cases), clavicle (4), spine (2), and sternum (1). In 73% of the patients in the study (27 individuals), the bone lesions existed in multiple sites like our case.

During the secondary phase of early-acquired syphilis, blood carries bacteria from the initial infection site to the periosteum, causing vascular inflammation. Such a process results in the formation of perivascular inflammatory infiltrations and granulation tissue rich in cells. The inflammation then spreads to the Haversian system within the bones, leading to osteitis and osteomyelitis [38]. Periostitis frequently manifests in this condition, as TP tends to adhere to the periosteum in the pathophysiological state at first. The spread of the inflammation to adjacent soft tissues could be considered a characteristic feature of osteosyphilis [39].

There have been cases reporting cranial osteosyphilis involving soft tissues [40, 41]. To the best of our knowledge, this is the first study to report that temporal arteritis was suspected because the inflammation extended to the temporalis muscle. Meningitis secondary to skull base osteosyphilis in an HIV-infected patient has also been reported [41]. Although the patient in this case was not HIV-infected, it is important to suspect that meningitis in association with osteomyelitis occurs even when the patient is HIV-negative, like in the current case. This case is also noteworthy for demonstrating an inflammatory process involving the extraosseous soft tissues and meninges.

Recent report indicate that 3D-FLAIR imaging [42] is useful for detection of subtle lesions, as in the present case. This imaging technique offers high spatial resolution that facilitates the identification of small lesions and provides excellent contrast for detecting abnormal intensities. Additionally, neurosyphilis can cause vasculitis in cerebral vessels; therefore, it is important to include the differential diagnosis in event of unexplained stroke occurrence in a young patient [43]. Recent advancements in vessel wall imaging [44] allow visualization of vascular wall inflammation, which might be useful in diagnosing vasculitis caused by syphilis and reflect vascular inflammatory activity.

Nevertheless, this study has several limitations. First, 3D-FLAIR images were not acquired due to the lengthy procedures required for their acquisition and thus, not routinely obtained. Use of these imaging techniques could have made it easier to depict lesions. Future studies are warranted to determine effectiveness of 3D-FLAIR in the detection of neurosyphilis lesions. Second, the use of contrast agents (Gd) in examinations makes a gumma in the internal auditory canal more clearly visible, although contrast enhancement material (Gd) was not used in the examination of case #2. This was because while Gd contrast agents are not contraindicated in pregnancy, their safety has not been fully established [45].

In conclusion, these three cases of syphilitic optic neuritis, otosyphilis, and osteoshyphilis underline the significance of including secondary syphilis in the list of differential diagnoses. Our findings suggest that each characteristic radiological feature, along with serological testing in even immunocompetent individuals, should prompt the consideration of a syphilitic infection. While uncommon, awareness of this condition can facilitate early diagnosis and offer proper treatment. Radiological imaging plays a pivotal role in these diagnoses to help avoid unnecessary biopsies.

## Data Availability

No datasets were generated or analysed during the current study.

## Declarations

### Abbreviations

CNS Central nervous system.

GCA Giant cell arteritis.

HIV Human Immunodeficiency Virus.

RPR Rapid Plasma Reagin.
